# Gauging speech and language markers of dementia against pathological and neuroimaging data

**DOI:** 10.1002/alz70857_097191

**Published:** 2025-12-24

**Authors:** Adolfo M. Garcia

**Affiliations:** ^1^ Global Brain Health Institute, University of California, San Francisco, CA, USA; Cognitive Neuroscience Centre, University of San Andres, Victoria, Buenos Aires, Argentina

## Abstract

**Background:**

Automated speech and language analysis (ASLA) is gaining momentum in the digital biomarker arena. Yet, limited attempts have been made to validate them against pathological and neurocognitive measures. Here I describe four studies correlating and comparing ASLA metrics with such measures in persons with Alzheimer's dementia (AD), mild cognitive impairment (MCI), and nonfluent/agrammatic variant primary progressive aphasia (nfvPPA).

**Method:**

Across studies, we assessed 60 persons with AD, 52 with MCI, 24 with nfvPPA, and 100 healthy controls (HCs). We extracted speech timing (e.g., pause duration, pause duration variability, articulation rate) and word property (e.g., frequency, semantic granularity, semantic variability) metrics from verbal fluency and paragraph reading tasks. We combined inferential statistics and machine learning to establish these metrics’ (a) sensitivity each disorder, (b) correlations with underlying brain changes, (c) diagnostic comparability with standard measures, and (d) robustness to predict autopsy‐confirmed pathology.

**Results:**

First, our fluency results from AD show that word property metrics robustly differentiate persons with AD from HCs (AUC = .89), while capturing temporo‐frontal atrophy and default‐mode‐network hypoconnectivity. Second, in a replication study incorporating speech timing features, we obtained similar classification results (AUC = .83), further showing that these outcomes do not differ significantly from those obtained via standard cognitive (AUC = .81) or neuroimaging (AUC = .89) features (*p*‐values > .05). A third study showed that similar results emerge when classifying persons with MCI and HCs (AUC = .80), surpassing cognitive measures (AUC = .71; *p* < .05) and predicting the volume of temporal brain regions typically affected by this disorder. Finally, a study on nfvPPA showed that speech timing metrics can consistently differentiate patients from HCs (AUC = .95), predicting both frontal atrophy patterns and autopsy‐confirmed pathology years before the patients’ death.

**Conclusion:**

Overall, the evidence suggests that ASLA metrics reveal early‐stage markers of AD and related conditions, predicting syndrome‐specific brain anomalies and underlying pathology. More importantly, results match the sensitivity of standard neuropsychological and neuroimaging measures, meeting the non‐inferiority criterion for emerging digital tests. Given the affordability and scalability of ASLA, these findings solidify their role as a tool to favor equity in dementia screenings and assessments.

